# Virtual reality cognitive remediation tool for individuals with mild cognitive impairment: study protocol for a feasibility randomized clinical trial

**DOI:** 10.3389/fpubh.2024.1477279

**Published:** 2024-11-22

**Authors:** Alessandra Perra, Diego Primavera, Valerio Leonetti, Roberta Montisci, Daniele Carta, Giulia Lorrai, Aurora Locci, Luchino Chessa, Angelo Scuteri, Giulia Cossu, Antonio Egidio Nardi, Lucia Valmaggia, Alessia Galetti, Valerio De Lorenzo, Federica Sancassiani, Mauro Giovanni Carta

**Affiliations:** ^1^Department of Medical Sciences and Public Health, University of Cagliari, Cagliari, Italy; ^2^Strategic Steering Commitee, Centro Studi SAPIS Foundation, Italian National Federation of Orders of Radiographers and Technical, Rehabilitation, and Prevention Health Professions Research Centre, Rome, Italy; ^3^University Hospital of Cagliari, Cagliari, Italy; ^4^Institute of Psychiatry, Federal University of Rio de Janeiro, Rio de Janeiro, Brazil; ^5^Orygen, Centre for Youth Mental Health, University of Melbourne, Parkville, VIC, Australia; ^6^Department of Psychology, King’s College London, Institute of Psychiatry, Psychology & Neuroscience, London, United Kingdom; ^7^Department of Clinical Psychiatry, Katholieke Universiteit Leuven, Leuven, Belgium; ^8^University of Tor Vergata, Rome, Italy

**Keywords:** mild cognitive impairment, cognitive remediation, virtual reality, psychiatric rehabilitation technicians, speech therapists

## Abstract

**Introduction:**

With the growing older adult population, the European Union emphasizes the need to promote research in healthy aging trough multidisciplinary and innovative approaches, including the integration of advanced technologies like virtual reality (VR) in cognitive rehabilitation. This reflects the increasing awareness of the importance of addressing challenges related to neurodegenerative diseases in the older adult population. Our study aims to present a protocol that will assess the feasibility and provide a preliminary measure of effectiveness for an intervention using immersive CR technology for cognitive remediation (CR) in individuals with Mild Cognitive Impairment (MCI).

**Methods:**

A feasibility randomized controlled clinical study will involve 30 individuals who are over 65 years old, both sex, who meet the diagnostic criteria for MCI from the University Hospital of Cagliari, randomly assigned to either the experimental condition or control group. Both groups will continue to receive standard pharmacological therapy. The experimental group will undergo a 3-months cognitive remediation program using fully immersive VR with two sessions per week. Each session will last a maximum of 60 min and will be supervised by expert health professionals. In contrast, the control group will continue with standard care. The intervention program will be carried out by s psychiatric rehabilitation technicians and speech therapists, emphasizing a comprehensive framework aligned with healthcare needs. Feasibility will be assessed based on tolerability, including dropout rates and acceptability, which considers the proportion of recruited participants among those considered eligible and on side effects and level of satisfaction. The preliminary measures of effectiveness will be evaluated on quality of life, cognitive functions, biological and social rhythms, depressive symptoms and anxiety.

**Results:**

The trial findings will be submitted for publication in international peer-reviewed journals and shared at international meetings and conferences.

**Discussion:**

This study aiming to assess the feasibility and preliminary effectiveness of a fully immersive VR/CR program for MCI in order to give data for a subsequent confirmatory trial. The results of the pilot RCT are expected to significantly contribute to research on the prevention of neurocognitive degeneration, with a specific emphasis on enhancing the application of technologies. The strengths of this work are the high technological innovation program for mental health treatments for *healthy aging* and multidisciplinary approach emphasizing a holistic framework aligned with health needs.

## 1 Introduction

As the global population continues to age, mild cognitive impairment (MCI) and dementia have become primary contributors to disability, particularly among the older adult population (1, 2). MCI delineates a transitional stage between the typical cognitive decline (CD) attributed to progressive aging and the initial phase of dementia (3, 4). Italy is the European country with the oldest population (23%), projections indicate that by the year 2050, one out of every five individuals in Italy will be in old age. This leads to a reduction in the working-age population and an increase in direct and indirect healthcare/social costs associated with the rising health problems (5). Risk factors such as physical, cognitive, and social inactivity are estimated to be responsible for about 40% of the risk of developing dementia over the lifespan (6). In general, approximately 50% of people with MCI progress to dementia within 3 years (7). Furthermore, CD is associated with depressive and anxiety symptoms and they both contribute to functional deterioration in daily tasks (7, 8). These conditions reduce the quality of life and the regulation of biological and social rhythms (9–11). Among others risk factors hypertension and cardiometabolic play a crucial role in the onset of cognitive dysfunction and progression to clinical over dementia (12). However, the pharmacological treatment and management of these conditions in older subjects is complex (13, 14) and may often result in worsening of cognitive progression (15). Growing attention has been given from researchers and from the European Union (EU) in recognizes the important goal of promoting research in the field of healthy aging, particularly non-pharmacological interventions aimed at preventing and/or delaying CD (16, 17).

Recent literature reviews have highlighted promising outcomes regarding cognitive training or cognitive remediation (CR) interventions and physical activity in improving cognitive performance in older adult individuals in an integrated public health approach, irrespective of CD (2, 18–21). Moreover, a substantial amount of evidence in the field of neuropsychological disorders, encompassing conditions such as dementia and MCI, has been documented (2, 22, 23). Cognitive training or cognitive remediation programs fall under the broader category of cognitive rehabilitation and utilize various cognitive-behavioral techniques. These treatments share key components, such as the identification of personal goals, the development of strategies to achieve them, and the application of these strategies to everyday life (20). Consequently, interventions should utilize behavioral techniques that effectively facilitate rehabilitation outcomes. Scientific evidence suggests that cognitive remediation interventions yield positive results in the treatment of MCI, both in terms of cognitive outcomes and overall functioning (2, 24). CR interventions are defined as behaviorally based interventions aimed at improving cognitive processes (memory, attention, executive functions, social cognition, and metacognition) with the goal of achieving persistence of results and their generalization (25–27).

The need to promote research in healthy aging trough multidisciplinary and innovative approaches, including the integration of advanced technologies like virtual reality (VR), in cognitive rehabilitation has also been emphasized by the EU (16, 17). This reflects the increasing awareness of the importance of addressing challenges related to neurodegenerative diseases in the older adult population. The use of VR as a CR tool has shown several scientific advancements in recent years (28–30). VR serves as a crucial tool facilitating the learning and/or enhancement of various skills, owing to its ability to make learning experiences more realistic and ecologically valid compared to traditional intervention techniques (31). Recent systematic reviews suggest that the utilization of innovative technologies, such as immersive VR, in the treatment of CD in MCI, may lead to positive outcomes (29, 32). These findings not only indicate improvements in cognitive functions (memory, language and executive function) but also achieve an overall clinical, personal and social functioning enhancement (33). Although there is positive evidence, the methodological quality still requires improvement. Frequently, studies lack detailed descriptions of the VR intervention and the methods employed during sessions. Moreover, the majority of the studies did not specify the framework for developing the intervention, which is crucial for explaining the coherence between hypotheses, outcomes, methods, and the games used in the VR program and the cognitive domains being trained (34). This lack of information hinders the establishment of a standard operating procedure, essential for the reproducibility of the intervention and the development of a future golden standard (29). It is therefore crucial to promote clinical trials implementing CR interventions with fully immersive VR programs. Specifically, training should target all cognitive functions aligned with the identified health needs for MCI, further promote the generalization of trained skills to everyday life (35, 36). Lastly, an approach centered on personal recovery goals and multidisciplinary is essential to address the complexity of emerging needs.

### 1.1 Aims

Given all these considerations, the hypothesis of the study is that a CR intervention using fully immersive VR in the older adult population with MCI may yield positive outcomes in terms of feasibility (acceptability, tolerability and side effects) and preliminary clinical outcomes (cognitive functions, quality of life, biological rhythms, anxiety and depression symptoms). The aim of this study is to presents the research protocol of a randomized controlled trial (RCT). The RCT is designed to evaluate the feasibility and preliminary clinical efficacy of a CR program through fully immersive VR for treating CD in MCI among individuals over 65 years of age.

## 2 Materials and methods

### 2.1 Study design

This study is a feasibility randomized controlled (two-arm) clinical study protocol, adhering to the CONSORT flow diagram extension for feasibility study (37). The protocol is written according to the SPIRIT Standard Protocol Items (38). The RCT will involve individuals aged 65 and above with MCI. The experimental group will participate in a three-month CR program utilizing fully immersive VR, consisting of two sessions per week in addition to standard care. In contrast, the control group will receive only standard care. Consistent with the study outcomes (primary outcome of feasibility and secondary outcome of preliminary efficacy measurement), a randomized controlled design is essential even at the feasibility stage to ensure greater scientific rigor. For both outcomes, the variables will be assessed through comparison between the two groups.

### 2.2 Participants

The RCT will involve individuals diagnosed with MCI, who will be recruited from the Cardiology and Internal Medicine departments at the University Hospital of Cagliari (San Giovanni di Dio Civil Hospital). The enrollment process will be conducted at the Consultation and Psychosomatic Psychiatry Center within the same University hospital. Eligible participants must meet the following inclusion criteria: age 65 and above, a diagnosis of Minor Neurocognitive Disorder (MND) specifically MCI based on DSM-V criteria (4), of any genders, and able to provide informed consent. Exclusion criteria include failure to meet inclusion criteria, a diagnosis of epilepsy or severe visual impairments due to potential risks associated with extensive virtual reality stimulation, and severe illnesses that hinder attendance at bi-weekly interventions.

### 2.3 Randomization

Participants meeting the eligibility criteria will be randomly assigned to two groups (intervention and control group). A computer-generated randomization list will be used for the random allocation sequence, carried out at the University of Cagliari. A standard single blinding randomization procedure will be employed without stratification; however, if the sample will not be homogeneous, block stratification by age and sex, with a maximum of two blocks, will be implemented. An external statistician, not involved in the research project (thus not involved in assessments or interventions), will generate the two lists using a list generator and before it will anonymize the allocation using identification codes. The researchers involved in the assessments (initial and post-intervention) will be blinded to the participants’ group assignments.

### 2.4 Blinding

Because of the intervention method, it may not be feasible to effectively blind both the participants and professionals engaged in the project.

### 2.5 Assessments

Patients will be screened and enrolled at the Consultation and Psychosomatic Psychiatry Center of the University Hospital of Cagliari (San Giovanni di Dio Civil Hospital) and will be assessed before the treatment (t0), immediately post-intervention (t1), and after 6 (t2) and after 12 (t3) months post-intervention. The researchers responsible for measuring the outcomes will be three, and they will not be involved in the intervention phase. The following socio-demographic variables will be collected for each participant: gender and age; marital status; educational background; past and current occupational status; past and current organic physical illnesses; past and current mental health diagnoses; medications in use.

#### 2.5.1 Primary outcomes

The primary outcome assesses the feasibility of the study, focusing on acceptability, tolerability (dropout rate), level of side effects and satisfaction. Variables in terms of acceptability, tolerability and satisfaction level will be assessed using an *ad-hoc* data sheet. Acceptability is measured as the proportion of recruited patients among those deemed eligible, while tolerability is assessed as the proportion of patients completing the trial intervention among those included.

The Italian adaptation of the Simulator Sickness Questionnaire (SSQ) will be used to record side-effects. The SSQ is a 16-item self-report questionnaire assessing the frequency of potential side effects related to the use of VR technologies, such as nausea, dizziness, headaches, eye fatigue, etc. (39).

#### 2.5.2 Secondary outcomes

The secondary outcomes related to the preliminary clinical trial efficacy encompass cognitive functions (including visuospatial abilities, attention, memory, verbal and semantic fluency, and executive function), and personal and social functioning (symptoms of anxiety and depression, quality of life, emotional awareness, social functioning, and regulation of biological rhythms).

##### 2.5.2.1 Personal and social functioning

The following self-report questionnaires will be administered:

- Short form health survey with 12 items (SF-12), investigating dimensions of wellbeing including perception of general health, mental health, emotional and social functioning, and work and social roles. The questionnaire is validated in Italian (40).
- Brief social rhythms scale (BSRS), assessing the level of regularity in sleep–wake and appetite biological rhythms, and social rhythms through 10 items. The questionnaire is validated in the Italian language (41).
- Generalized anxiety disorder–7 item (GAD-7), based on DSM IV diagnostic criteria for generalized anxiety disorder, investigating the presence and severity of 7 anxiety symptoms in the previous 2 weeks, such as nervousness, inability to stop worrying, excessive worry, restlessness, difficulty relaxing, irritability, fear that something terrible will happen. The questionnaire is validated in Italian (42).
- Patient health questionnaire-9 items (PHQ-9), a questionnaire based on DSM IV diagnostic criteria for Major Depressive Disorder, investigating the presence and severity of 9 depressive symptoms in the previous 2 weeks, such as depressed mood, anhedonia, sleep and appetite disturbances, low energy, difficulty concentrating, motor behavior disturbances, loss of self-esteem, suicidal ideation; the cut-off for minor depressive disorder is represented by scores ≥5, and for major depression by scores ≥10. The questionnaire is validated in Italian (43).

##### 2.5.2.2 Cognitive functions

They will be evaluated by:

- Addenbrooke’s cognitive examination (ACE-R), a brief cognitive test with an administration duration of approximately 15 min in intact subjects, evaluating five cognitive areas: attention/orientation, memory, verbal fluency, language, visuo-spatial abilities. The test, with a maximum score of 100 and a cut-off for mild cognitive impairment of 66, includes the Mini Mental State Examination (MMSE). The test is validated in Italian (44).
- Matrices test, assessing indicators of selective attention (45).
- Rey’s word test, in both versions, assessing long-term memory, semantic memory, and specifying the phase of the mnemonic process in which the deficit occurs (46).
- Trail making test (TMT), in both versions, assessing cognitive functions such as cognitive flexibility, inhibitory control, and attention (47).
- Digit span normal and reverse, in both versions, assessing verbal short-term memory and working memory, respectively (48, 49).
- Stroop test, assessing selective attention, inhibition of irrelevant information, and cognitive flexibility (50).
- Frontal assessment battery (FAB), screening battery for global executive function through a series of cognitive and behavioral tests (51).
- Cognitive estimation test (CET), in both versions, assessing the ability for estimation and abstraction (52).
- Rey’s figure test, assessing praxis skills, visuo-spatial abilities, working memory, long-term memory, and executive functions (50).

### 2.6 Intervention

The experimental group will participate in an immersive VR-based CR program utilizing the “CEREBRUM” software version 3.0.1 developed by “PRoMIND-Services for mental health Srls” in collaboration with “IDEGO-Virtual Psychology.” CEREBRUM is among the latest VR-implemented CR tools in psychiatric rehabilitation and was designed by clinicians and experts specializing in cognitive rehabilitation, is compatible with the hardware “Oculus Quest” VR viewer all-in-one headset, a CE-marked device developed by Meta Technologies.

The CEREBRUM app provides 52 exercises of varying difficulty divided by Memory and Learning, Cognitive Estimates, and Attention and Working Memory modules. During VR exposure, participants do not actively interact with the virtual environments themselves. Instead, the clinician guides the session by asking structured questions, following a manual, allowing the participant to engage with the scenario through their responses. This approach ensures that the exposure is not solely dependent on interaction with the virtual reality, but rather on the dialog facilitated by the clinician. The virtual scenarios offer a fully immersive exposure simulating everyday reality, including home and urban environments. The intervention includes 24 sessions over 3 months, with two sessions per week lasting 50 min each. Each session includes reception, psychoeducation and orientation to the tool, exercise psychoeducation, psychoeducation to the function targeted in the exercise, generalization phase, execution of the VR exercise with feedback (one of attention and working memory module), post-exercise comment, a second exercise (one of Memory and Learning or Cognitive Estimation modules) following the same structure (maximum VR exposure duration of 15–20 min), final comment, and homework. Some sessions could include a third exercise based on participant, session, and operator’s assessment. The generalization phase refers to the explanation of the function and its significance in the participants’ life context. For homework, practical suggestions are intended to be implemented by the patients in their daily life. A multidisciplinary team composed of one psychiatric rehabilitation technician and a speech language therapist will be involved in each session, emphasizing a comprehensive framework aligned with the healthcare needs of the participants. The selection of this intervention methodology, aiming to enhance the generalization of cognitive and personal functioning through a diversity of trained domains, aligns with the health needs of individuals with MCI. To ensure the generalization and standardization of the intervention, the operators involved are professionals working in rehabilitation. They undergo specific training for the tool and follow a precise intervention manual during the session. The only variations during the session occur in the psychoeducation and generalization phases, where personal examples from the participant are used.

### 2.7 Control

The control group will include participants enrolled from the San Giovanni di Dio Civil Hospital and will receive conventional treatment, including general pharmacological and non-pharmacological interventions dependent on the health conditions of participants aged from 65 with MCI.

### 2.8 Data analyses

The statistical analysis will use SPSS software (version 21). Intra-rater and inter-rater reliability for the evaluation of the outcomes will be measured using Kappa statistics. Descriptive statistics for sociodemographic variables and the level of satisfaction of the experimental intervention, will be presented as frequencies (percentages) or mean ± standard deviation. To assess homogeneity between the experimental and control groups regarding “sex” and “age” distributions, chi-square tests and one-way ANOVA will be employed. A multivariate analysis, one for each considered outcome, will be conducted to compare means between the intervention and non-intervention groups over time (pre-and post-intervention), applying Bonferroni’s correction. The normality assumption of dependent variables will be assessed for sphericity, with Mauchly’s test use to verify the equality of variances among all combinations of related groups. Missing data will be considered incomplete and therefore excluded from the analysis.

### 2.9 Sample size considerations

In the registered clinical trial, our protocol outlined the recruitment and randomization of 30 eligible subjects in accordance with the predefined inclusion criteria.

### 2.10 Trial status and ethical aspects

The study is registered on ClinicalTrials.gov (NCT06270966), and approval has been obtained from the Regional Ethical Committee (Protocol VR AND MCI n. 28,287–08/11/2023). Currently, the recruitment phase for the study is about to commence.

## 3 Results

The results will concern the feasibility and preliminary clinical efficacy of a CR intervention using a fully immersive VR program for individuals with MCI. The trial results will be published in international peer-reviewed journals and disseminated at international congresses and meetings.

## 4 Discussion

### 4.1 Resource identification initiative

The primary objective of the trial is to comprehensively assess feasibility, particularly focusing on recruitment and retention rates. This evaluation provides essential insights for determining sample sizes in subsequent confirmatory trials. Additionally, to assess the side effect and satisfaction level of a VR-implemented CR tool, known as CEREBRUM. The secondary objective is to investigate cognitive and psychosocial variables. The hypothesis suggests that utilizing a CR with fully immersive VR program, with ecological instruments, can enhance cognitive abilities as well as other social and personal outcomes. The aim of this protocol study is to report the protocol of the trial, underlining methodological aspects in order to provide a response to the emerging health needs in line with the new evidences.

Indeed, the implementation of VR as a rehabilitative treatment in individuals with MCI is yielding positive results. Particularly, in a recent meta-analysis involving only people with MCI, a positive effect on clinical cognitive outcomes, specifically memory, language, and global cognition, was observed (29). However, few results are available regarding the improvement of clinical and functional outcomes due to the heterogeneity of variables and tools used, and the quality of the studies still need to improve (29, 53, 54). In general, the use of technological innovation and tools such as VR, based on the evidence described, can make the rehabilitation experience more engaging and facilitate the generalization of skills to real-life contexts, as the scenarios closely resemble those encountered in everyday life (54, 55). Naturally, technology should not replace the importance of the therapeutic relationship; rather, these are valuable tools that support rehabilitative techniques, with the relationship with the therapist remaining fundamental. Therefore, as in the intervention methodology used, exposure to VR is time-limited within each session.

Finally, for the present trial the purposed methodology uses a technological innovation tools that can more easily transfer the trained skills into the real world while always considering an approach based on the therapeutic relationship (31). The methodological approach employed in the trial reflects a person-centered and recovery-oriented approach to rehabilitation (29, 35, 36, 56), enabling participants to improve their skills and achieve an overall enhancement in their health and psychological wellbeing. Moreover, it aligns with the new framework for developing complex interventions where ensuring the reproducibility of interventions is a crucial methodological consideration (34, 57). Despite the methodological strength of the methodology, the present study is designed primarily as a feasibility study. Subsequent studies intending to evaluate efficacy and cost-effectiveness will require with a larger sample size. The findings of the trial will be deliberated among all the authors.
